# A Low-Frequency Fiber Bragg Grating Acceleration Sensor Based on Spring Support and Symmetric Compensation Structure with Flexible Hinges

**DOI:** 10.3390/s24102990

**Published:** 2024-05-08

**Authors:** Lijun Meng, Panpan Zhu, Xin Tan, Xiao Huang

**Affiliations:** 1 State Key Laboratory of Precision Blasting, Jianghan University, Delta Lake Road, No. 8, Wuhan 430056, China; 2 Intelligent Manufacturing College, Jianghan University, Delta Lake Road, No. 8, Wuhan 430056, China; 221227006047@stu.jhun.edu.cn (P.Z.); tanpatrick@jhun.edu.cn (X.T.); 3 College of Information Engineering, Wuhan Business University, No. 816 Dongfeng Boulevard, Wuhan 430056, China; 20200117@wbu.edu.cn

**Keywords:** vibration measurement, fiber Bragg grating, acceleration sensor, flexure hinge

## Abstract

To measure vibration signals, a low-frequency fiber Bragg grating (FBG) acceleration sensor featuring a flexible hinge with a spring support and symmetric compensation structure has been designed. Based on the mechanical model of the sensor’s structure, the expressions for sensitivity and resonant frequency of the sensor are derived. The structural parameters of the sensor are optimized, and a simulation analysis is conducted using ANSYS 19.2 software. According to the results of simulation analysis and size optimization, the sensor prototype is constructed. Subsequently, its amplitude-frequency response, sensitivity, and temperature characteristics are investigated through vibration experiments. The experimental results show that the resonant frequency of the sensor is 73 Hz, the operating frequency range is 0~60 Hz, and the sensitivity measures 24.24 pm/g. This design meets the requirements for measuring vibration signals at low frequencies.

## 1. Introduction

Vibration is a common physical phenomenon, such as the vibration of buildings caused by typhoons or other external factors, and the mechanical equipment running in industry will also have vibration. Building vibration [1,2] can lead to structural deformation, thereby compromising the safety of the structures. Similarly, vibrations in mechanical equipment [3,4] can result in damage or injuries; therefore, monitoring these vibrations is crucial to minimizing losses and enhancing safety. One of the key pieces of equipment for acquiring vibration signals is the acceleration sensor, which is widely used in the monitoring of large structures and for ensuring environmental safety. Compared to traditional electrical acceleration sensors, as an advanced optical fiber-sensing technology, fiber Bragg grating technology offers high sensitivity, a wide frequency response range, low noise, and strong resistance to electromagnetic interference, making it a research hotspot in the field of acceleration sensors [5,6,7,8].

Over the past three years, scholars both domestically and internationally have conducted a series of studies on fiber Bragg grating (FBG) acceleration sensors. The common structures of fiber Bragg grating (FBG) acceleration sensors primarily include cantilever beam, diaphragm, elastic cylinder, tube, and homotypic types [9,10,11]. Parida, O. P. et al. (2019) [12] designed a double-L cantilever fiber Bragg grating accelerometer. This model exhibits a resonant frequency of 86 Hz, sensitivity of 406.7 pm/g, linearity of 99.86%, and temperature sensitivity of 0.016 pm/°C. Zhang, F. et al. (2021) [13] proposed a compact structure dual fiber Bragg grating (FBG) vibration sensor, consisting of a dual diaphragm and a H-shaped hinge, which has a sensitivity of 32 dB re pm/g, a fluctuation of less than ±3 dB, and a resonant frequency above 1.6 kHz. Li, T. et al. [14] designed a diaphragm-based fiber Bragg grating acceleration sensor with a temperature compensation capability. Experimental results show that the acceleration sensitivity of the sensor is 20.189 pm/g, and it has an operational bandwidth of 10−200 Hz and a resonant frequency of 600 Hz. Within the temperature range of 30–90 °C, the temperature sensitivity is 8.66 pm/°C. Current sensors enhance the sensing sensitivity of fiber Bragg grating (FBG); however, they often encounter issues such as excessive structural size relative to their dimensions, inadequately compact structures, and susceptibility to grating chirping.

Therefore, the flexure hinge is a flexible and reversible transmission structure that offers advantages such as compact size, ease of processing, stable movement, and high precision. Researchers are increasingly exploring the use of flexure hinge structures in fiber grating sensing technology. Le, H.-D et al. (2023) [15] developed a medium-to-high frequency fiber Bragg grating (FBG) accelerometer based on a circular bending hinge structure. The sensor recorded a resonant frequency of approximately 1700 Hz and a sensitivity of 23 pm/g, with an operational frequency range of 500–1400 Hz. Li, Z et al. (2021) [16] designed a medium- and high-frequency dual fiber Bragg grating (FBG) vibration sensor utilizing a straight circular hinge. The resonant frequency of the sensor is approximately 2800 Hz, with a sensitivity of 21.8 pm/g within a flat frequency range of 50–1000 Hz. Recently, Luo, X et al. (2022) [17] designed a fiber Bragg grating (FBG) accelerometer based on a symmetrical double flexural hinge structure. This device has a resonant frequency of approximately 890 Hz and a sensitivity of about 41 pm/g; additionally, it is also capable of measuring vibrational acceleration within a broad range of 50–600 Hz. Although these sensors have a wide measurement frequency range, they are not suitable for low-frequency measurements. In another study, Wang, H et al. (2021) [18] proposed a composite bending hinge three-dimensional accelerometer. In the three spatial dimensions, the sensitivities of the sensor are 51.9, 39.5, and 20.3 pm/g, respectively, with resonant frequencies of 800, 1125, and 1750 Hz. The dimensions of the sensor are merely 25 mm × 25 mm × 30 mm. Song, H et al. (2020) [19] developed a two-dimensional fiber Bragg grating vibration sensor based on an orthogonal bending hinge structure. The resonant frequencies in the X and Y directions are approximately 1275 Hz and 1482 Hz, respectively, with a third-order natural frequency of 4932 Hz. The sensitivities in the X and Y directions are 41.2 pm/g and 34.5 pm/g, respectively. This design affords the sensor high torsional stiffness and robust resistance to torsional vibrations. However, these two structural designs are relatively complex and difficult to manufacture. Liang, L et al. (2022) [20] developed a miniature anti-bending fiber Bragg grating accelerometer featuring a flexible hinge design. This model exhibits a resonant frequency of approximately 900 Hz and a sensitivity of 26.962 pm/g. Li Y. et al. (2022) [21] also designed a high-sensitivity fiber Bragg grating sensor based on a flexible hinge that achieves a sensitivity of 804 pm/g at 15 and 30 Hz. Since temperature has a certain impact on the performance of fiber Bragg grating, although these two sensors perform well, the lack of temperature compensation can affect the overall measurement accuracy of the sensors. Li H. et al. (2022) [22] designed a multi-stage flexible hinge structure to reduce the stiffness of the flexible hinges. The resonant frequency of the entire fiber Bragg grating accelerometer is 84.2 Hz, and the sensitivity to external vibration signals is 927.21 pm/g. Nguyen, T.T.-V. et al. (2023) [23] proposed a symmetric dual-mass block fiber Bragg grating vibration sensor with a V-shaped flexible hinge. The sensor has an average sensitivity of 73.86 pm/g, a natural frequency of 800 Hz, and an operational range of 20–340 Hz. Both sensor types feature innovative flexible hinge designs; however, their use in extensive engineering projects is challenged by the lack of elastic buffering, which raises concerns about their durability and consistent long-term performance.

This paper introduces the design of a low-frequency fiber Bragg grating acceleration sensor that features a spring-supported and symmetrically compensated flexible hinge structure. This sensor is specifically developed for applications such as mechanical damage detection and monitoring of large structures in low-frequency environments. Additionally, FBGs are sensitive to temperature changes. Some existing FBG acceleration sensors do not take into account the impact of environmental temperature variations in the measurement signals, making it difficult to accurately capture the vibration signals of the objects being tested. Its symmetrical flexible hinge design facilitates temperature compensation, minimizing measurement inaccuracies, while the spring mechanism provides a buffering effect that enhances the sensor’s long-term operational stability.

## 2. Sensor Structure Design and Theoretical Analysis

### 2.1. Sensor Structure Design

**Figure 1:**
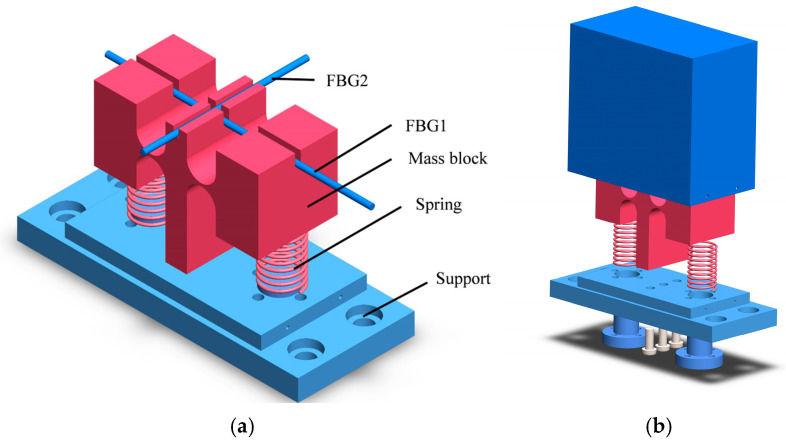
Schematic diagram of the sensor structure: (**a**) schematic diagram of sensor structure; (**b**) sensor modeling explosion view translation.

The structure of the sensor is depicted in Figure 1. The sensor consists of a T-shaped support column, a spring, a flexographic hinge, a mass block, and a base, all of which are organized into three main parts. The mass block is integrated with the T-shaped support column. At its base, the column is anchored to the base via three threaded holes. The lower part of the spring connects to the base using a nut, while the upper part is attached to the mass block at a pre-designated spring positioning hole using an adhesive. The left and right ends of the sensing fiber Bragg grating (FBG1) are secured within specially reserved slots for grating paste using AB glue. This grating area is strategically located in a groove above the T-shaped support to minimize motion interference and lateral displacement of the fibers during the vertical oscillations of the mass blocks. Additionally, FBG2, used for temperature compensation, is affixed in a separate groove designed to monitor changes in ambient temperature.

When the sensor is subjected to external vibrations, the mass block swings around the flexible hinge. This movement drives the axial strain effect on the sensing fiber Bragg grating (FBG1) attached to the upper part of the sensor, resulting in a drift in the central wavelength of the grating. Both mass blocks move in the same direction. Meanwhile, FBG2 is tasked solely with measuring the temperature changes during the experiment, providing necessary temperature compensation for FBG1.

### 2.2. Theoretical Analysis of Sensors

**Figure 2:**
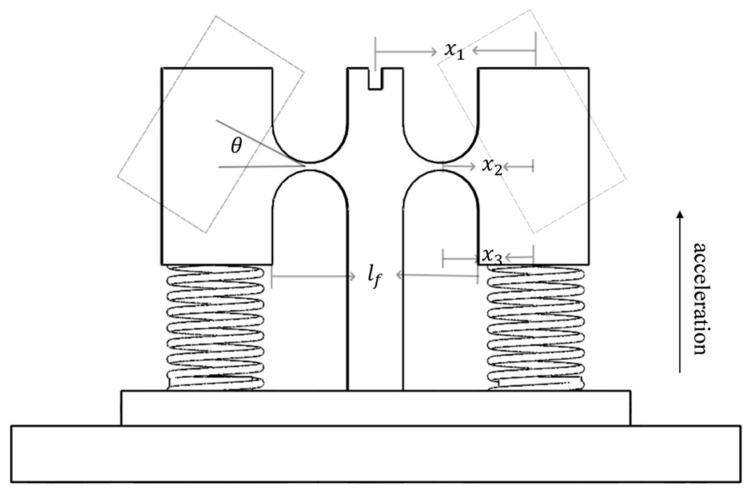
Mechanical model of the sensor.

The changes in the *Z*-axis stress structure of the sensor are depicted in Figure 2. Given the complete symmetry of the structure, it is sufficient to analyze the mass block on one side.

Here, let m represent the total mass of the mass block. The acceleration is set to a(t)=Asin(ωt), A is the amplitude of the acceleration in the *z*-axis direction, while ω is the angular frequency of the external excitation.

It can be obtained that the torque balance formula of the whole system is

(1) $$J\overset{..}{\theta }+\left[K_{z}+K_{f}x_{1}^{2}+K_{g}x_{2}^{2}\right]\theta =mx_{3}\mathrm{Asin}(\omega t)$$

where J is the moment of inertia of the mass block, θ is the rotation Angle of the mass block, $K_{z}$ is the hinge stiffness, $K_{f}$ is the grating stiffness coefficient, $x_{1}$ is the distance from the center of the FBG1 gate region to the center of rotation, $K_{g}$ is the spring stiffness coefficient, $x_{2}$ is the distance from the center of the spring to the center of rotation, and $x_{3}$ is the distance from the center of the mass block to the center of rotation. In addition, the hinge of this sensor is a straight circular flexure hinge, and the entire structure is deformed by the force on the axis, as shown in Figure 2. Therefore, the rotational stiffness of the flexure hinge around the Z-axis is

(2) $$K_{z}=\frac{{\mathrm{Etr}}^{2}}{12V}$$

Among them [24,25],

(3) $$V=\left[\frac{2s^{3}(6s^{2}+4s+1)}{(2s+1){\left(4s+1\right)}^{2}}+\frac{12s^{4}(2s+1)}{{\left(4s+1\right)}^{\frac{5}{2}}}\arctan\sqrt{4s+1}\right]$$

s is a variable, $s=\frac{r}{t}$, E is the elastic modulus of the flexure hinge material, t is the hinge width, and r is the hinge arc radius.

According to Equation (1), the resonant frequency of the sensor in the *z*-axis direction can be determined

(4) $$F=\frac{1}{2\pi }\sqrt{\frac{K_{z}+K_{f}x_{1}^{2}+K_{g}x_{2}^{2}}{J}}$$

Since the rotation Angle of θ is very small in the actual vibration process and the relationship between the amount of wavelength change at the center of the FBG and the strain can be expressed as

(5) $$\Delta \lambda =(1-P_{e})\cdot \lambda \cdot \varepsilon$$

Here, λ is the initial central wavelength of FBG1, Δλ is the central wavelength shift, ε is the strain of FBG1, and $P_{e}$ is the effective optical fiber elastic coefficient.

It can be obtained that the maximum drift of the center wavelength of the grating is

(6) $$\Delta \lambda =(1-P_{e})\cdot \lambda \cdot \frac{2x_{1}}{l_{f}}\cdot \frac{mx_{2}A}{\left[K_{Z}+k_{f}x_{1}^{2}+k_{g}x_{2}^{2}-J\omega^{2}\right]}$$

Here, $l_{f}$ is the dangling effective length of the gate region.

Therefore, the sensor sensitivity is known as from the FBG principle

(7) $$S=\frac{\Delta \lambda }{A}=2(1-P_{e})\lambda \frac{mx_{2}x_{3}}{l_{f}\cdot \left[K_{Z}+k_{f}x_{1}^{2}+k_{g}x_{2}^{2}-J\omega^{2}\right]}$$

It can be observed from the formula that the central wavelength of the sensing grating varies with the amplitude of the detected vibration signal; therefore, the amplitude characteristics of the vibration signal can be inferred by measuring the grating signal. When the frequency and amplitude of the detected vibration signal are constant, there is a linear relationship between the change in the center wavelength of the grating and the amplitude of the vibration signal, given a specific flexible hinge structure and sensing grating. Consequently, the flexure hinge FBG sensor can determine the intensity of vibrations based on the variation in signal strength at the grating’s center wavelength.

## 3. Structure Simulation

### 3.1. Analysis of Structural Parameters

According to the theoretical formula analysis, the flexure hinge radius (r), hinge width (t), and mass (m) significantly influence the natural frequency and sensitivity of the sensor. Under certain conditions, the sensitivity and resonant frequency of the sensor are inversely related. For instance, as the hinge width (t) increases, the sensitivity of the acceleration sensor decreases while the overall resonant frequency increases. To design a sensor of appropriate size that meets measurement requirements, it is essential to analyze the structural parameters of the sensor. The size of the mass block notably affects the overall mass, dimensions, and sensing characteristics of the sensor. Therefore, the effects of four primary parameters—flexure hinge radius (r), hinge width (t), mass block height (h), and mass block thickness (w)—on the sensing characteristics are primarily simulated and analyzed. Based on these findings, the sensor’s structural size is optimized. MATLAB is utilized to analyze these key parameters (r, t, h, and w). The sensor and the experimental platform are constructed from 304 stainless steel, while the preliminarily selected sensor dimensions, along with the test grating FBG1 parameters, are presented in Table 1.

**Figure 3:**
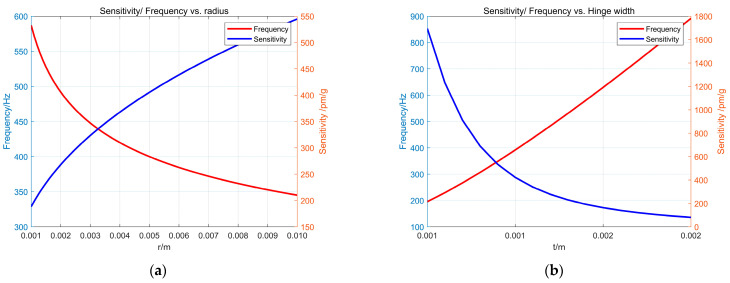
The influence of hinge radius r and hinge thickness t on sensitivity and resonant frequency: (**a**) the influence of r on sensitivity and resonant frequency; (**b**) the influence of t on sensitivity and resonant frequency.

When the thickness of the mass block w = 30 mm, the height of the mass block h = 28 mm, and the radius and width of the hinge vary in a range from 1 mm to 10 mm and 0.5 mm to 2 mm, respectively, the variation curves of the resonant frequency and sensitivity of the sensor obtained according to Equations (4) and (7) are shown in Figure 3a,b. The sensitivity of the sensor increases with the increase in the flexure hinge radius r and decreases with the increase in the hinge width t. The natural frequency is vice versa, and the size of the sensor is limited, so r = 5.5 mm is selected. Considering the difficulty of physical processing, t = 1 mm is selected.

**Figure 4:**
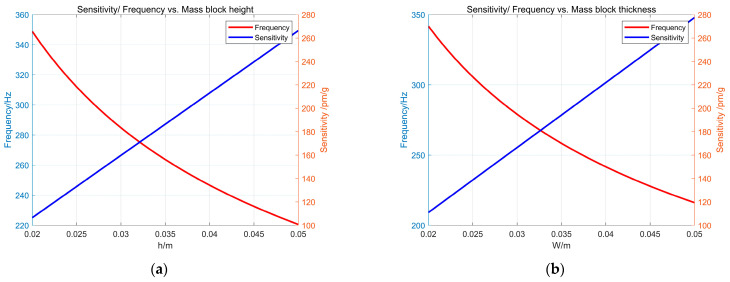
The influence of the mass block height (h) and mass block thickness (w) on the sensor’s sensitivity and resonant frequency is detailed as follows: (**a**) the impact of the mass block height (h) on both sensitivity and resonant frequency; (**b**) the impact of the mass block thickness (w) on both sensitivity and resonant frequency.

When the hinge radius r = 5.5 mm, the hinge width t = 1 mm, and the height and thickness of the mass block vary in a range from 20 to 50 mm, the resonant frequency and sensitivity of the sensor are obtained according to Equations (4) and (7). Figure 4a,b shows that the sensitivity of the sensor increases with the increase in the mass block width W and the mass block height H. The resonant frequency decreases as W and h increase. It is necessary to meet the measurement requirements of the sensor body but also consider the overall size of the sensor body and the difficulty of installation, so w = 30 mm and h = 28 mm are selected.

### 3.2. Finite Element Analysis

**Figure 5:**
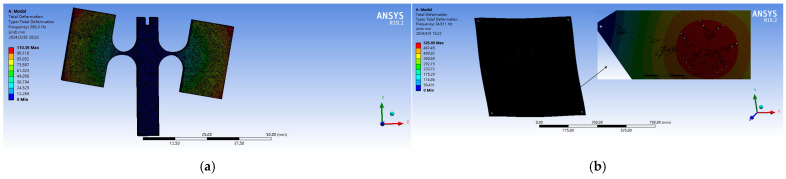
Finite element simulation diagram: (**a**) Sensor modal simulation; (**b**) Plate modal simulation.

To analyze the vibration sensing characteristics of the sensor, it is modeled using SolidWorks. Given that the experimental platform is also made from 304 stainless steel—which may resonate with the sensor—a finite element analysis is conducted for both the sensor and the platform. The two models are then imported into ANSYS, where modal simulation analyses of both the sensor structure and platform structure are performed using ANSYS WORKBENCH. Initially, the structural material performance parameters are set as shown in Table 1. The simulation results for the sensor are displayed in Figure 5a and those for the flat panel are shown in Figure 5b.

According to the simulation results, the resonant frequency of the sensor’s first mode is 386.3 Hz, which exhibits a slight deviation from the theoretical calculation of 406 Hz. This discrepancy may be attributed to processing errors. The third mode shape has a resonant frequency of 1421.5 Hz, significantly higher than that of the main mode shape, indicating that the sensor possesses good lateral anti-interference capabilities. The resonant frequency of the first mode of the plate is 24.5 Hz; therefore, it is crucial to focus on analyzing experimental data around 24 Hz when collecting data in the experiment.

## 4. Experimental Results and Analysis

**Figure 6:**
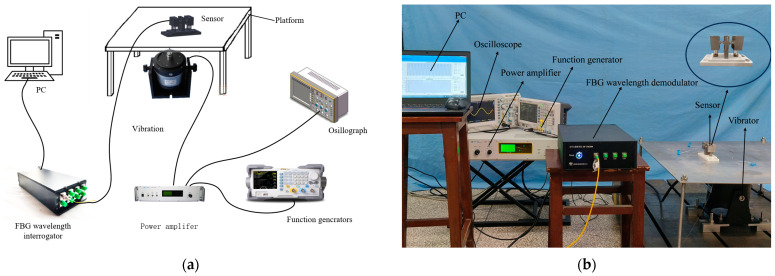
Sensor testing experimental system: (**a**) schematic diagram of the Experimental System; (**b**) experimental system physical image.

The vibration experiment system, as shown in Figure 6, comprises several components, including a function generator, power amplifier, oscilloscope, FBG demodulator, shaker, experimental plate, and computer. The function generator sends a signal to the power amplifier, which then amplifies the current signal and activates the shaker. This prompts the plate to undergo regular forced vibrations up and down. The flexure hinge FBG sensor is securely bolted onto the platform and vibrates synchronously with the platform at the same frequency. When the acceleration sensor detects the vibration, the reflection spectrum changes. The demodulator processes this change and transmits the signal to the computer, which displays and records the data in real-time.

### 4.1. Analysis of Output Response Characteristics

**Figure 7:**
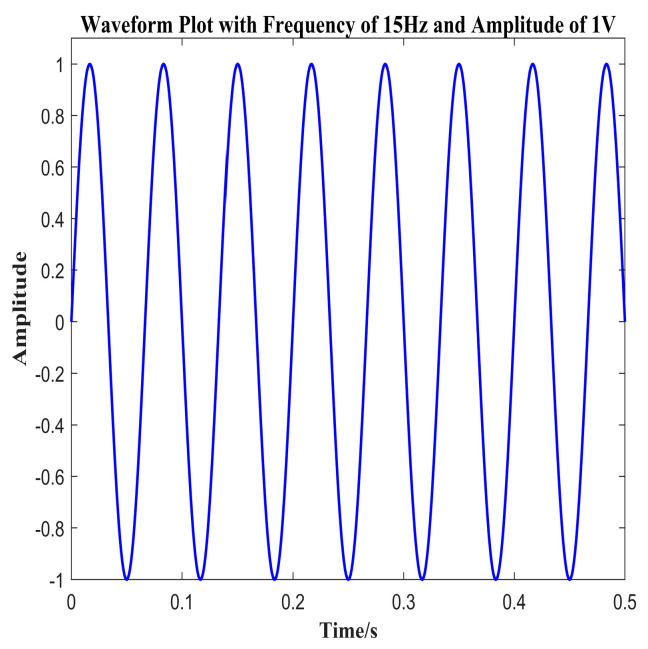
Signal generator output waveform.

In our experiments, we used a commercial fiber Bragg grating demodulator to measure the changes in the FBG center wavelength over time. The demodulator, manufactured by O-OPTICS in China, model OP-FBG500, maintains a measurement precision of 1 pm and a demodulation speed of 8000 Hz, meeting the practical measurement requirements for minor changes and slow variations in the grating center wavelength. Additionally, to reduce the impact of high-frequency noise on the measurement signal, we applied low-pass filtering to the signals from the demodulator. The vibration exciter and power amplifier used in the experiment are DH40100 and DH5872 of DongHua Testing Technology Co., Ltd. respectively. The DH40100 is a unidirectional inertial vibration exciter, and its output inertial force has a linear relationship with the input current. According to the shaker’s output characteristic curve, the current signal at the control input stabilizes around 2A. Consequently, the force exerted on the center of the exciting aluminum plate is approximately maintained at 20N during the experiment. The output wavelength signal of the vibration sensor under forced vibration is then obtained. The 15 Hz sine wave signal, triggered by the signal generator, is depicted in Figure 7.

**Figure 8:**
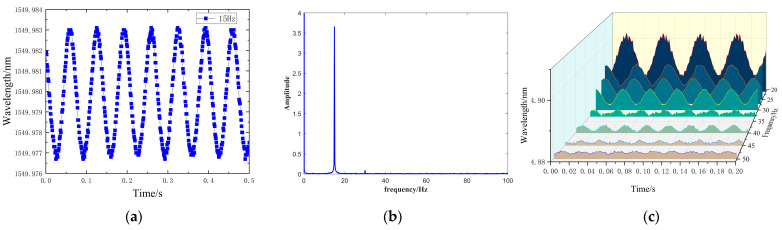
Sensor output response curve: (**a**) 15 Hz waveform plot; (**b**) 15 Hz amplitude spectrum. (**c**) Waveform plots at different frequencies.

Figure 8 demonstrates that the overall time-domain waveform is well-formed, indicating that the sensor effectively captures the external signal and accurately reflects the external sinusoidal excitation. Furthermore, spectrum results obtained through a Fourier transform confirm that the vibration frequencies measured by the sensors are consistent with those of the external excitation. This consistency underscores the sensors’ excellent detection performance within their respective operating frequency ranges.

### 4.2. Analysis of Amplitude and Frequency Characteristics

The natural frequency of an FBG (fiber Bragg grating) vibration sensor primarily relates to the sensor’s physical and mechanical structure. When external vibrations approach or reach the sensor’s natural frequency, the sensor will resonate, meaning its vibration amplitude at this frequency significantly increases. At this time, due to the increased strain, the wavelength of light in the fiber Bragg grating changes. This wavelength shift can be precisely measured with a fiber optic demodulator, thereby determining whether the frequency of the external vibration detected by the sensor is close to or equal to its natural frequency.

**Figure 9:**
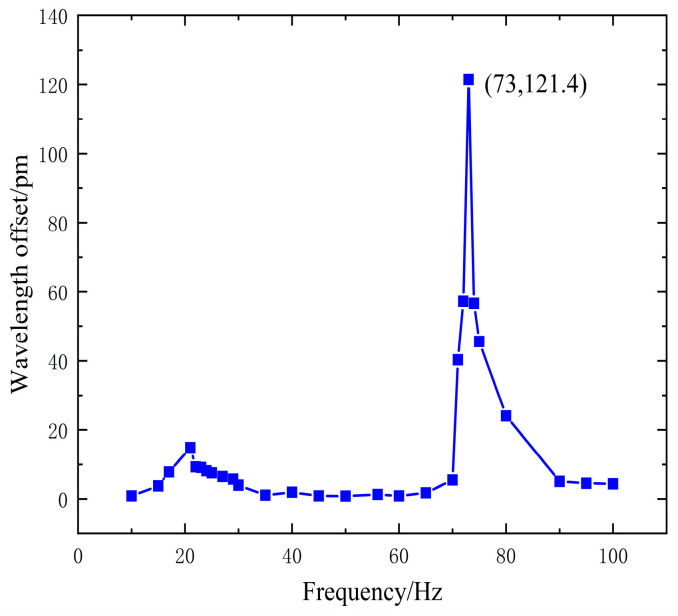
Sensor amplitude-frequency response characteristics.

To assess the sensing response characteristics, the output of the power amplifier was set to a constant 1A, thereby fixing the shaker’s output signal amplitude at 10 N. A frequency sweep experiment was then conducted on the FBG acceleration sensor, starting at 5 Hz and increasing in increments of 3 Hz before narrowing to 1 Hz as it approached the resonant frequency. The wavelength variations were recorded using an FBG wavelength demodulation instrument, and the results are displayed in Figure 9.

During the experiment, severe vibrations and resonances were observed in the experimental platform when the signal frequency reached 20 Hz, indicating that the actual natural frequency of the plate was approximately 20 Hz. This finding deviates from the theoretical predictions, likely due to variations in plate manufacturing. Additionally, the resonant frequency of the sensor was measured at 73 Hz, different from the simulated 73 Hz. This discrepancy can be attributed to the experimental setup, where the single hinge structure was mounted on the experimental platform using a 3D-printed base. Such mounting influenced the sensor’s behavior, altering its working range and resonant frequency from the simulated values.

When the frequency of the input signal is too high, exceeding the demodulation capability of the fiber optic demodulator or the designed response range of the sensor, high-frequency signals may not be accurately restored. Therefore, if the frequency exceeds this range, the accuracy and sensitivity of the sensor could significantly decrease, especially at frequencies higher than its natural frequency. As such, the sensor can only measure vibration signals with frequencies below its natural frequency. As shown in Figure 8, the response is relatively flat within the range of 0–60 Hz, indicating that the operational frequency range of the sensor is 0–60 Hz.

### 4.3. Sensitivity and Linearity Test

In the experiment, the shaker is directly connected to the platform. When the power amplifier outputs 1A, the amplitude of the shaker’s output excitation is 10 N. Given that the mass of the plate is 9.35 kg, it can be deduced using Newton’s second law, F=ma, that the acceleration of the plate is 1.07 m/s^2^ when the power amplifier outputs 1A.

**Figure 10:**
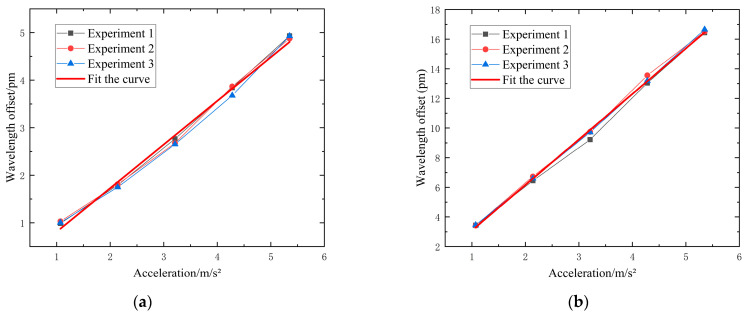

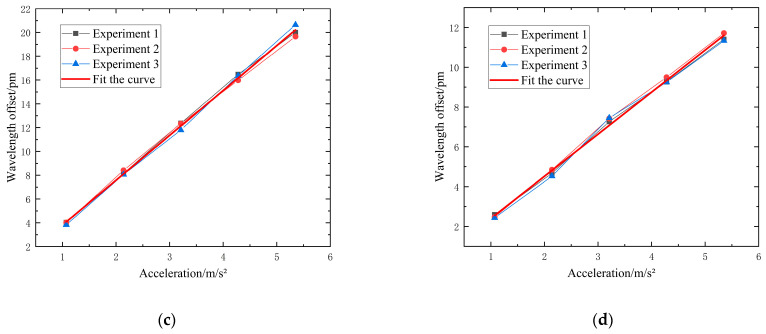
Calibration experiment of FBG acceleration sensor: (**a**) 10 Hz; (**b**) 15 Hz; (**c**) 30 Hz; (**d**) 40 Hz.

The sensitivity of the FBG acceleration sensor was determined by calculating the ratio of the FBG wavelength shift to the platform’s acceleration. The experimental platform was subjected to vibration excitations at frequencies of 10 Hz, 15 Hz, 30 Hz, and 40 Hz, with power amplifier outputs ranging from 1A to 5A. The experimental results, replicated three times for consistency, are displayed in Figure 10.

Table 2 indicates that all sensitivity calibration curves exceed 0.99, demonstrating good linearity. In conclusion, the overall sensitivity of the sensor, when connected to the plate, is approximately 24.24 pm/g. This value significantly deviates from the theoretical prediction of 82.9 pm/g. The discrepancy arises because the excitation is applied directly to the plate and only indirectly sensed by the sensor body. The theoretical analysis was based on a standalone sensor body structure where the mass block is directly subjected to force, leading to a substantial difference between the theoretical and experimental outcomes.

Three experiments were conducted, each under controlled conditions. According to Table 3, when the output of the power amplifier was set to 2A and the function generator emitted signal excitations at frequencies of 10 Hz, 15 Hz, 30 Hz, and 40 Hz, respectively, the repeatability error was recorded at 2.03%. This low error percentage indicates good repeatability of the experimental results.

### 4.4. Temperature Interference Test

**Figure 11:**
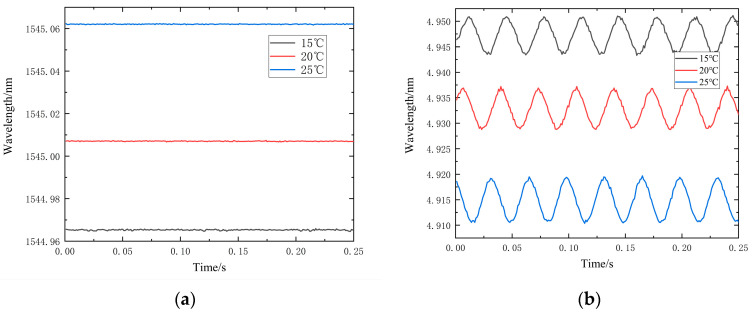
Wavelength variation plot of FBG at different temperatures: (**a**) FBG2 wavelength variation plot; (**b**) FBG1-FBG2 wavelength variation plot.

Given that temperature affects the central wavelength of the fiber Bragg grating, an experiment was conducted to assess the impact of various temperatures on the sensor’s characteristics. To accomplish this, the overall temperature of the laboratory was varied to observe the effects on the central wavelength shift of FBG1 while the sensor concurrently received a vibration signal. Figure 11 displays results when the power amplifier output was set to 2A and the function generator’s output frequency was 30 Hz.

According to Table 4, the wavelength offset for a single FBG is measured at 9.6 pm/°C. However, after differential processing of FBG1 and FBG2, the wavelength offset reduces significantly to only 0.27 pm/g, approximately 2.8% of that observed in a single FBG. These results clearly demonstrate that the dual FBG structure effectively mitigates the impact of temperature variations on the sensor’s measurements. Table 5 flexure hinge acceleration affected by temperature compared to existing low-frequency tests" needs to be added

## 5. Conclusions

This paper introduces a low-frequency fiber Bragg grating acceleration sensor with a flexible hinge (supported by springs and featuring a symmetric compensation structure) aimed at addressing the challenges of measuring low-frequency vibration signals. The resonant frequency and sensitivity of the sensor are theoretically analyzed, with parameters being optimized using MATLAB R2018b software and simulations being conducted using ANSYS software. Theoretical calculations suggest that the resonant frequency of the single sensor structure is 383.6 Hz and the sensitivity is 82.9 pm/g, which meet the measurement requirements. Experimental results, however, indicate a natural frequency of 73 Hz and a sensitivity of 24.24 pm/g under conditions where the excitation device is directly connected to the experimental platform but not directly to the sensor. Although these results satisfy the measurement requirements, improvements in processing technology and material purity are necessary. Enhancing these aspects will help to miniaturize the entire sensor structure and reduce errors.

## Figures and Tables

**Table 1:** Parameters of FBG acceleration sensor.

Name	Value/Unit
Center wavelength of fiber Bragg grating	1550/nm
Elastic-optic coefficient of fiber Bragg grating	0.22
Cross-sectional area of fiber Bragg grating	1.23 × 10^−8^/m^2^
Elastic modulus of fiber Bragg grating	72/GPa
Effective length of suspended region in the grating	5/mm
Elastic modulus of the sensing element	190/GPa
Density of the sensing element	7850/kg/m^3^
Poisson’s ratio of the sensing element	0.3
Length of the mass block	15/mm

**Table 2:** Sensitivity of the sensor at different frequencies.

Frequency/Hz	Slope of the Fitted Curve	R^2^	Sensitivity/pm/g
10	0.982	0.9924	9.01
15	3.287	0.9984	30.16
30	4.035	0.9996	37.02
40	2.262	0.9967	20.75

**Table 3:** FBG wavelength drift at different frequencies.

Frequency/Hz	Wavelength Shift/pm				Standard Deviation/pm
	Experiment 1	Experiment 2	Experiment 3	Average	
10	1.794	1.810	1.750	1.785	0.03
15	6.45	6.74	6.595	6.595	0.12
30	8.161	8.415	8.060	8.212	0.15
40	4.671	4.844	4.526	4.680	0.13

**Table 4:** Wavelength variation in FBG at different temperatures.

	Temperature/°C	15	20	25
FBG2	Wavelength/nm	1544.965	1545.007	1545.061
FBG1-FBG2	Wavelength offset/pm	7	8	9.1

**Table 5:** Performance comparison of flexible hinge accelerometers for low-frequency testing.

Reference	Resonant Frequency/Hz	Sensitivity/pm/g	Flat Region/Hz	Temperature Compensation/pm/°C
[14]	600	20.189	10–200	8.5/
[20]	900	26.962	20–400	--
This paper	73	24.24	0–60	0.27

## Data Availability

Data are contained within the article.
